# Clinical and Pathological Changes in Rams Experimentally Infected with *Actinobacillus seminis* and *Histophilus somni*

**DOI:** 10.1155/2014/241452

**Published:** 2014-01-27

**Authors:** Valéria S. Moustacas, Teane M. A. Silva, Luciana F. Costa, Custódio A. Carvalho Júnior, Renato L. Santos, Tatiane A. Paixão

**Affiliations:** ^1^Departamento de Clínica e Cirurgia Veterinárias, Escola de Veterinária, Universidade Federal de Minas Gerais, Avenida Antônio Carlos, 6627, 31270-901 Belo Horizonte, MG, Brazil; ^2^Departamento de Patologia Geral, Instituto de Ciências Biológicas da Universidade Federal de Minas Gerais, Avenida Antônio Carlos, 6627, 31270-901 Belo Horizonte, MG, Brazil

## Abstract

Infectious epididymitis is considered a major cause of economic losses for the sheep industry worldwide. This study aimed to investigate clinical and pathological changes associated with experimental infections with *A. seminis* and *H. somni* in rams. Twenty rams of age 18 to 24 months were infected by intraepididymal inoculation of *A. seminis* (*n* = 10) and *H. somni* (*n* = 10). Rams were weekly examined and biological samples were collected during six weeks. All rams inoculated with *A. seminis* and 80% inoculated with *H. somni* became infected. The recovery of bacteria was possible in semen and urine samples and tissues in both experimental groups. Clinically, there were a decrease in testicular consistency and an increase in measures of the left epididymis tails in both experimental groups. The main gross changes were observed in the reproductive tract. Microscopically, the main lesions were inflammatory changes in the genitourinary tract and testicular degeneration. *A. seminis* and *H. somni* were able to colonize several organs of the genitourinary tract in rams, being indistinguishable by clinical exam, necropsy or histopathology. For differential diagnosis, it is important to use diagnostic techniques for direct confirmation of the etiologic agent.

## 1. Introduction

Ovine infectious epididymitis is one of the major causes of reproductive disorders in sheep, and it strongly impacts on the productivity of the herds [1]. The losses are due to lower fertility rate, reduction in the number of birth, and early culling of breeders [2, 3]. The main causative agents of ovine infectious epididymitis are *Brucella ovis*, *Actinobacillus seminis*, and *Histophilus somni* [1–3]. Ovine brucellosis caused by *B. ovis* is considered the most important cause of ovine infectious epididymitis in the world [4]. This disease has been reported in all major sheep producing regions in the world, including Brazil. *A. seminis* is a natural inhabitant of the preputial mucosa of young sheep [5] and it can act as an opportunistic pathogen, causing primarily epididymitis and orchitis in young animals [6–8]. *H. somni* is also considered an opportunistic pathogen, residing naturally in the preputial mucosa of rams [5], and reproductive and respiratory mucous membranes of cattle [9]. However, while many studies have been performed in reference to *H. somni* infection in cattle as reviewed by Corbeil (2007) [9], little has been investigated about this infection in rams [10–12].

Rams affected by infectious ovine epididymitis may develop epididymal and/or testicular asymmetry, with increased consistency of the epididymis and decreased consistency of ipsilateral testis. In most chronic cases, testicular consistency tends to increase, reflecting the atrophy and fibrosis, independent of involved etiologic agent [3, 13]. Several previous studies evaluated the pathological changes in rams experimentally infected with *B. ovis *[14–16]. In contrast, there are only a few studies about lesions caused by *A. seminis* [8, 17] and *H. somni* [12]. Thus, considering the importance of ovine infectious epididymitis and the lack of studies relating to infections with *A. seminis* and *H. somni* in rams, the aim of this study was to characterize the lesions caused by each agents and their distribution in the reproductive tract of experimentally infected rams.

## 2. Material and Methods

Twenty crossbred Santa Inês rams ranging from 18 to 24 months of age were used in this study. All rams were confirmed as free of infectious epididymitis by *B. ovis*, *A. seminis*, and *H. somni*, by clinical examination, agar gel immunodiffusion test for *B. ovis*, and bacteriological culture of semen, urine, and blood samples for isolation of these three agents. The rams were divided into two groups of 10 animals each and the experiments were carried out in Belo Horizonte, Brazil (19.52° S, 43.57° W). They were fed hay and commercial ration throughout the experiment. Both groups underwent two months of adaptation and training for semen collection using an artificial vagina. For semen sampling, a crossbred ewe had estrus induced with 2 mg of estradiol cypionate (ECP-Pfizer Animal health, Brazil) intramuscularly 48 hours before semen sampling. This protocol was repeated whenever necessary.

After the adaptation phase, the first group of 10 rams was inoculated with 1 mL of suspension containing approximately 2.3 × 10^10^ colony-forming units (CFU)/mL of *A. seminis* (strain ATCC 15768) injected into the left epididymis tail [18]. The second group of 10 rams was inoculated with 1 mL of suspension containing approximately 1.0 × 10^9^ CFU/mL of *H. somni* (strain 3384Y) also injected into the left epididymis tail [18]. Experimental infections were conducted consecutively and the experimental groups never had contact. The place was decontaminated with broom fire and utensils used to feed, handle, or collect biological samples of the animals were decontaminated by sterilization or with hypochlorite solution prior to use in other group. Biological samples obtained from both experimental infections were used to diagnosis proceedings [18]. The experimental protocols have been approved by the Committee of Ethics in Animal Experimentation (CETEA-UFMG, Protocols 285/2008 and 002/2010).

The rams were evaluated once prior to inoculation and every seven days of postinoculation (dpi), during six weeks, totaling seven evaluations by the experimental group. Clinically, scrotal circumference was measured in the testicular region of larger diameter with a measuring tape. The testicular consistency was assessed by palpation and assigned score of 1 (very flaccid and inelastic) to 5 (hardened). The length and width of the tail of epididymis were measured with caliper. The length was measured in dorsoventral and width in craniocaudal direction. All clinical evaluations were carried out by the same examiner.

To confirm the infection, semen, urine, and blood were obtained prior to inoculation and every seven dpi, during six weeks. To avoid cross-contamination between semen samples from different rams in each group, we used a sterile, disposable plastic inside the artificial vagina, connected directly to collection tube. Rams that did not show libido at some point of the trial period were subjected to electroejaculation [13]. Whole blood was collected by jugular vein puncture with vacuum collection system. Urine collection was performed by blocking the breath for 30 seconds. After six weeks of infection, the animals underwent euthanasia after sedation with xylazine 2 mg (Copazine-Schering-Plough Coopers, Brazil) followed by electrocution. For the determination of tissue distribution of agents, fragments of tail, body and head of the epididymes, testicles, ampullae of the vas deferens, seminal vesicles, bulbourethral glands, kidneys, bladder, inguinal and iliac lymph nodes, spleen, and liver were collected. For microbiological analysis, tissue samples were placed in 50 mL tube containing 2 mL of sterile PBS and macerated with a homogenizer. Fragments of the same tissues were fixed by immersion in 10% buffered formalin solution for subsequent histological processing. Fragments of testicles were also fixed in Bouin's solution.

For *A. seminis* isolation, 100 *μ*L of each sample (tissue homogenates, semen, blood, and urine) was plated in GC medium (chocolate Agar base medium, Bectron Dickinson, USA), supplemented with 1% bovine hemoglobin (Sigma-Aldrich, Brazil), and incubated at 37°C for 48 hours. *H. somni* was isolated using the same medium supplemented with 0.5% yeast extract (Invitrogen, Brazil) and incubated under an atmosphere with 5% CO_2_. Colonies were confirmed after resuspension in 100 *μ*L sterile ultrapure water, boiled for 10 minutes and the DNA amplified by species-specific PCR assay for each agent [19–21].

Histopathological evaluation was performed after tissue processing by the routine paraffin embedding and staining with hematoxylin and eosin (HE). Inflammatory changes were scored as follows: 0 (absent), 1 (mild), 2 (moderate), and 3 (severe). *A. seminis* antigen was detected in tissues with histological changes by immunohistochemistry. Polyclonal serum anti-*A. seminis *or anti-*H. somni* produced in rabbits was used as primary antibodies in streptavidin-peroxidase protocol as previously described [22].

Normally distributed data (scrotal circumference) were analyzed by ANOVA followed by Dunnett's multiple comparisons test. Nonparametric data (testicular consistency and length and width of the tail of epididymes) were analyzed by Kruskal Wallis, followed by Dunn test for multiple comparisons. The frequencies of positive samples for *A. seminis* and *H. somni* were compared by Fisher's exact test. Differences were considered statistically different when *P* < 0.05. The analyses were performed with the GraphPad InStat program, version 3.05 (GraphPad Software, Inc. InStat, USA).

## 3. Results

**Figure 1:**
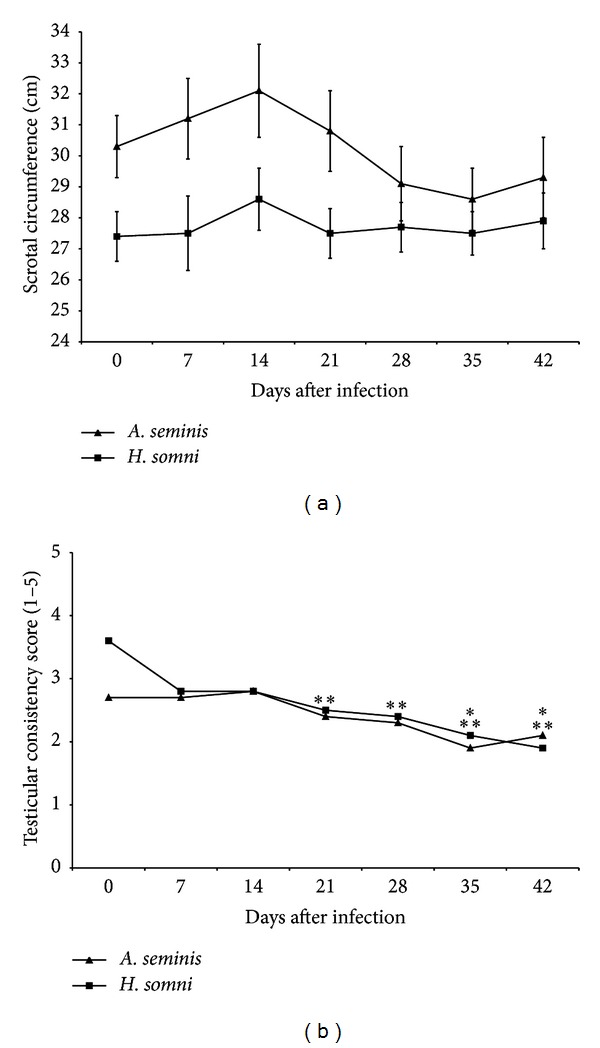
Testicular parameters of rams experimentally infected with *Actinobacillus seminis* (*n* = 10) or *Histophilus somni* (*n* = 10) during 42 days of infection. (a) Scrotal circumference. The values of each point represent the average and standard error. (b) Testicular consistency. The values of each point represent the mean. Asterisks indicate statistical difference (*P* < 0.05) when compared to preinfection values (time 0) by Dunn test for multiple comparisons (*A. seminis; **H. somni).

**Figure 2:**
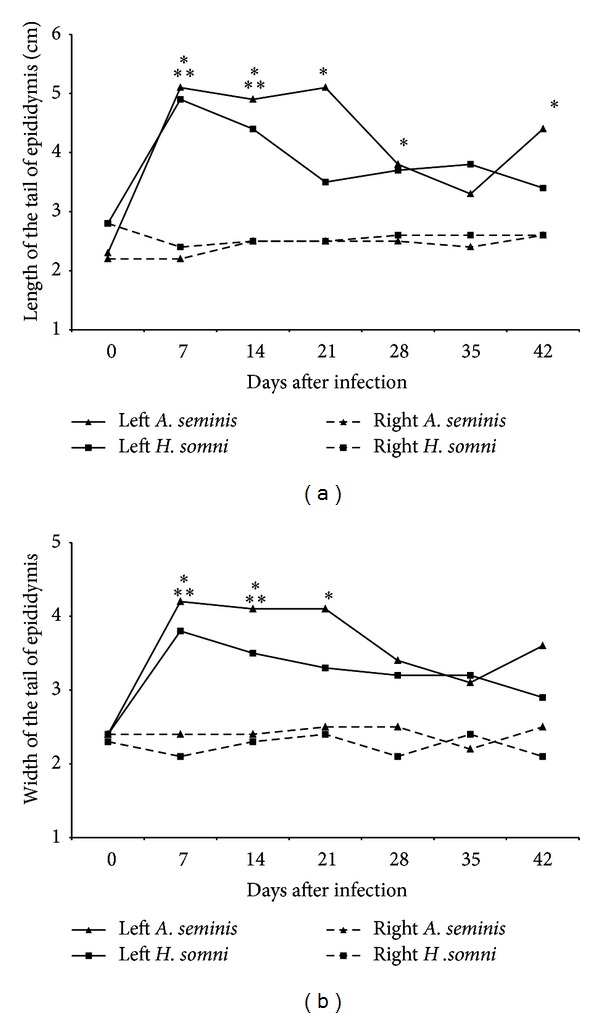
Length (a) and width (b) of the tail of the epididymides of rams experimentally infected with *Actinobacillus seminis* (*n* = 10) or *Histophilus somni* (*n* = 10) during the course of infections. Asterisks indicate statistical difference (*P* < 0.05) when compared to preinfection by Dunn test for multiple comparisons (*A. seminis; **H. somni).

Prior to inoculation, all 20 rams were clinically healthy, without palpable lesions in the testes or epididymides. Additionally, all rams were negative for infections with *B. ovis*, *A. seminis*, and *H. somni* by bacteriology of semen, urine, and blood. Throughout the experimental period, there was no change of scrotal circumference in both experimental groups (Figure 1(a)). However, the testicular consistency was decreased at 35 dpi, remaining decreased for the rest of the experimental period, in rams infected with *A. seminis*, while the group infected with *H. somni* had decreased testicular consistency as early as 21 dpi, also remaining decreased until the end of the experimental period (Figure 1(b)). Measurements of the right tail of the epididymides remained unchanged throughout the course of the experimental infections (Figure 2). However, the measurements of the left tail of the epididymides (i.e., inoculation site) significantly increased (*P* < 0.05) at 7 dpi in both infections. Rams infected with *A. seminis* had the length of the left epididymides tail increased up to 28 dpi, returning to a value similar to preinoculation time point at 35 dpi and then increasing again at 42 dpi (Figure 2(a)). The width remained increased up to 21 dpi, returning to the original values to 28 dpi (Figure 2(b)). The length and width of the left epididymides tails of rams *H. somni* infected returned to original size at 21 dpi (Figures 2(a) and 2(b)).

**Figure 3:**
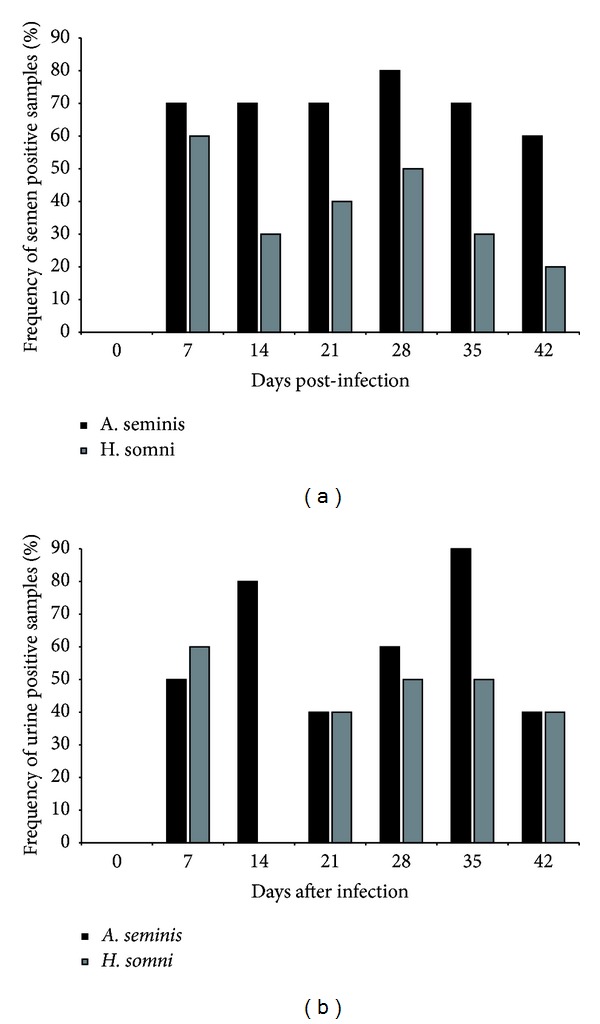
Frequency (%) of positive rams for bacterial isolation of *Actinobacillus seminis* (*n* = 10) or *Histophilus somni* (*n* = 10) over 42 days of experimental infection. (a) Semen and (b) urine.

Intraepididymal *A. seminis *inoculation resulted in infection of all 10 rams, since the agent was recovered by bacterial isolation from samples from all inoculated rams, in at least one time point during the course of infection. In contrast, inoculation with *H. somni* resulted in infection of eight of 10 inoculated rams. The frequency of positive rams by bacteriology over the experimental period is presented in Figure 3. It was possible to recover bacteria in semen and urine samples as early as 7 dpi (Figure 3). In addition, frequency of detection of *A. seminis* in semen and urine samples was higher (*P* < 0.05) than that of *H. somni* (Table 1). Neither *A. seminis* nor *H. somni* was isolated from blood samples.

**Figure 4:**
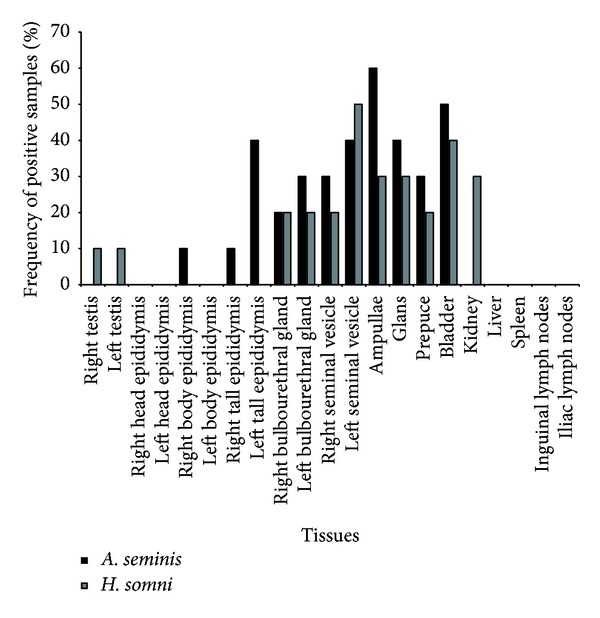
Frequency (%) of positive rams for bacterial isolation of *Actinobacillus seminis* (*n* = 10) or *Histophilus somni* (*n* = 10) in tissues after 42 days of experimental infection.

Seven (7/10) and five (5/10) rams infected with *A. seminis* and *H. somni*, respectively, had at least one organ positive by bacteriology. While *A. seminis* was recovered mainly from ampullae (60%) and urinary bladder (50%), *H*.* somni *was more often isolated from the left seminal vesicle (50%) and urinary bladder (40%, Figure 4). Additionally, *H. somni* was isolated from kidneys of 30% of infected rams. Nevertheless, the frequency of positive tissues was similar between both groups. Interestingly, the recovery of both agents at the inoculation site was low, with 40% left epididymis tail from *A. seminis*-infected rams bacteriologically positive and no positive cultures from the left epididymis tail from *H. somni*-infected rams. There was no bacterial recovery from inguinal and iliac lymph nodes, spleen, and liver.

**Figure 5:**
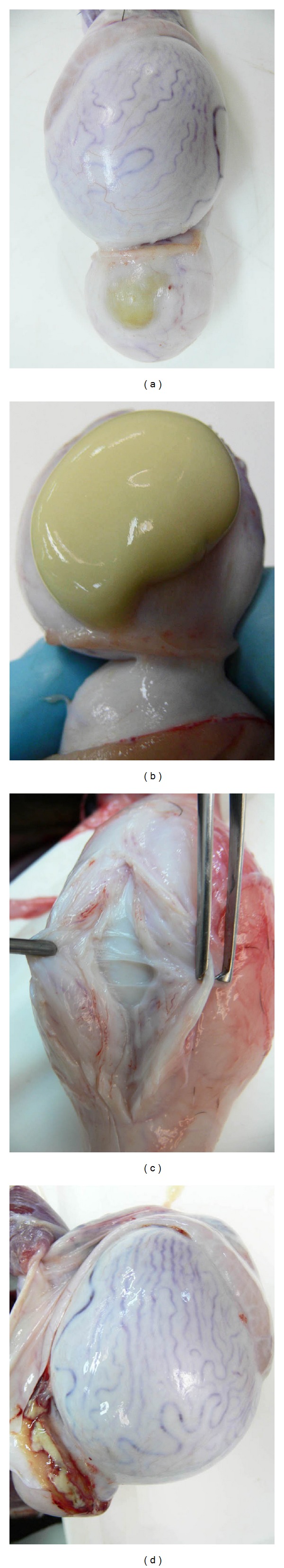
Gross findings in rams experimentally infected with *Actinobacillus seminis.* ((a) and (b)) Epididymal abscess. (a) Tail of the left epididymis is increased with focal yellowish area and (b) filled with yellowish viscous fluid (purulent exudate). (c) Testicular atrophy is associated with diffuse fibrosis of the tunica vaginalis. Left testis is decreased in volume and tunica vaginalis is thickened and firmly adhered to the testis. (d) Severe purulent epididymitis. Tail of the left epididymis is surrounded by purulent exudate and hemorrhage.

**Figure 6:**
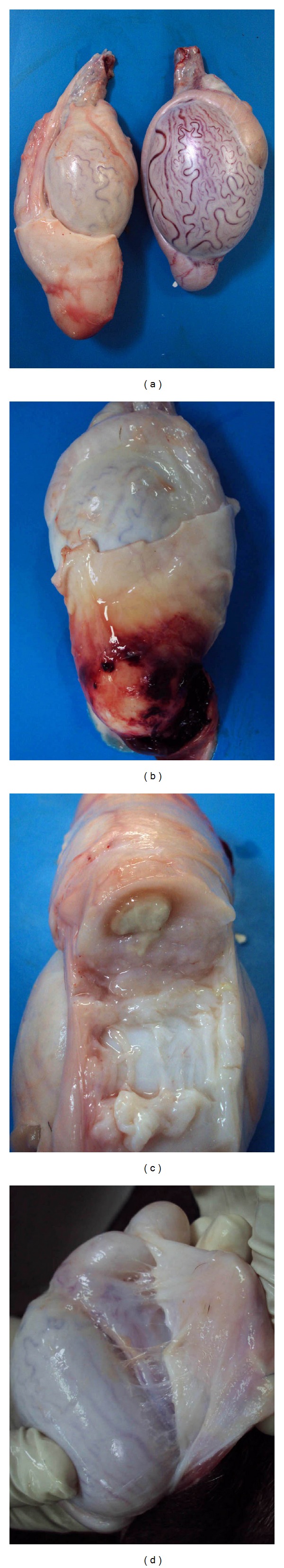
Gross findings in rams experimentally infected by *Histophilus somni*. ((a) and (b)) Unilateral epididymitis associated with testicular atrophy. (a) The body and tail of left epididymis are severally increased in size with tunica vaginalis adhered and ipsilateral testis strongly decreased. (b) Hematoma in tunica vaginalis adjacent to the tail of the epididymis. (c) Epididymal abscess. Body of left epididymis is increased in size and focal area in cut surface with yellowish viscous material. (d) Chronic periorchitis. Multifocal fibrous adhesions between the tunica albuginea and the tunica vaginalis.

Rams infected with *A. seminis* had macroscopic lesions located only in the reproductive tract. Changes frequently observed include abscess in the left epididymis tail, which was the site of inoculation (Figures 5(a) and 5(b)), and an increase in size of inguinal and iliac lymph nodes (Table 2). The abscesses ranged from 0.5 to 5.0 cm in diameter. The left testis had reduced size and consistency, and in one ram atrophy of the left testis associated with diffuse fibrosis of the tunica vaginalis was observed (Figure 5(c)). One ram had purulent exudate and diffuse hemorrhage around the tail of the left epididymis (Figure 5(d)). Similarly, rams inoculated with *H. somni* presented macroscopic changes especially in the genital tract. The main lesions observed were an increase in volume and abscesses in the tail of the left epididymis (Figure 6(a)). Hematoma adjacent to tunica vaginalis was observed in the tail of the left epididymis from one ram (Figure 6(b)). Abscesses were also observed in the body of the left epididymis (Figure 6(c)). Thickening and fibrous adherence of tunica albuginea with tunica vaginalis were also frequent changes (Figure 6(d)). Other macroscopic changes observed are listed in Table 2.

**Figure 7:**
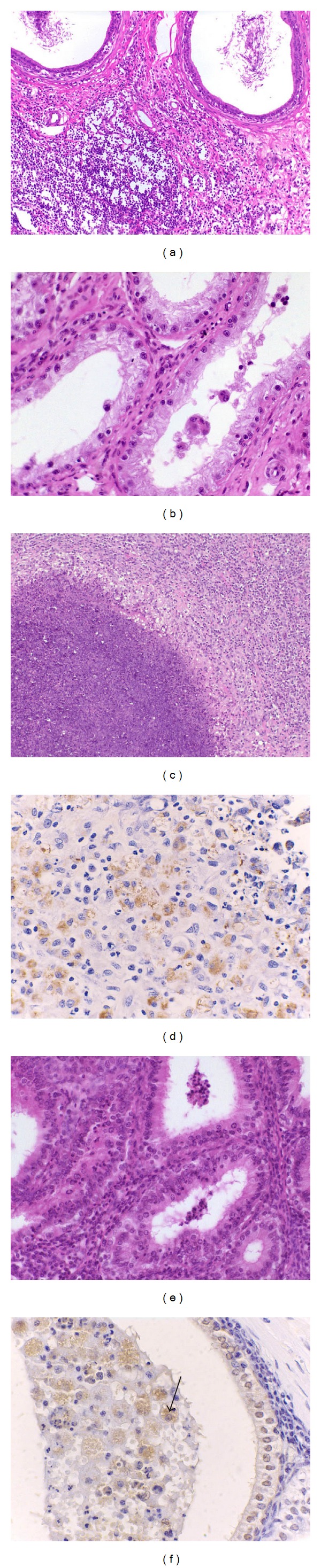
Microscopic findings in rams experimentally infected with *Actinobacillus seminis*. (a) Tail of the epididymis, focal severe mononuclear interstitial infiltrate, HE 200x. (b) Testis, intense degeneration with reduction of seminiferous epithelium layers, HE 400x (c) Tail of the epididymis, sperm granuloma, HE 200x. (d) Tail of the epididymis, and immunostained bacteria within the cytoplasm of cells in the granuloma, streptavidin-peroxidase 600x. (e) Seminal vesicle, interstitial and intraluminal inflammatory infiltrate, HE 400x. (f) Seminal vesicle, immunostained bacteria within the cytoplasm of macrophages in glandular lumen (arrow), streptavidin-peroxidase 600x.

**Figure 8:**
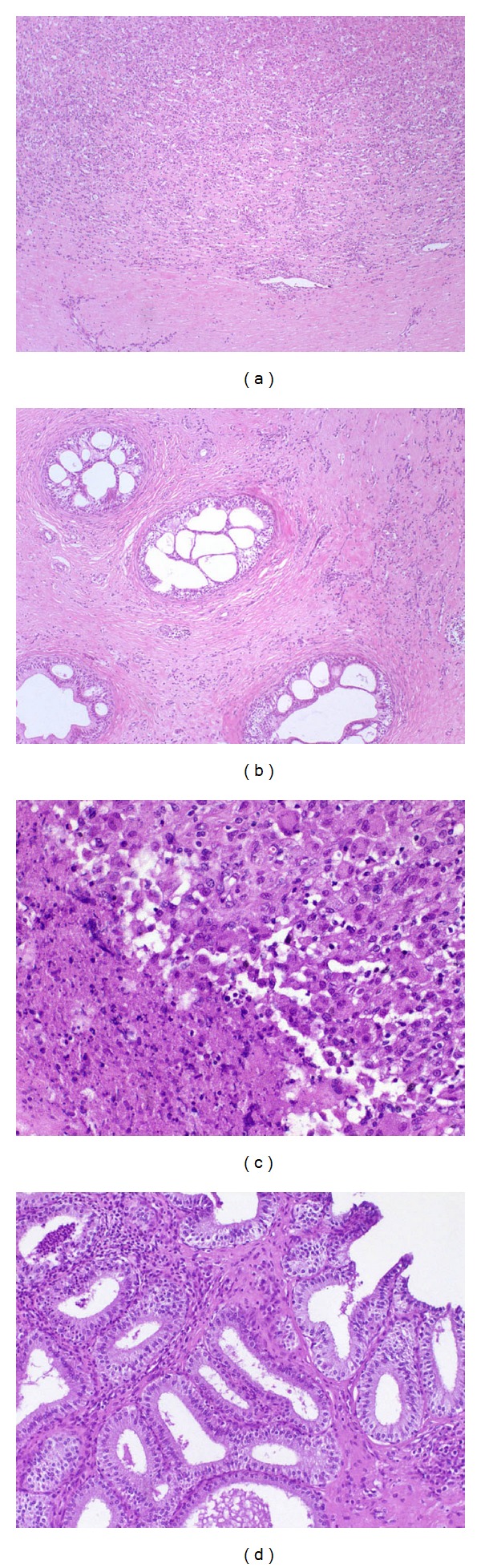
Microscopic findings in rams experimentally infected by *Histophilus somni*. (a) Tail of the epididymis, diffuse mononuclear infiltrate, HE 100x. (b) Tail of the epididymis, multiple cysts in the ductal epithelium and chronic mononuclear infiltrate, HE 100x. (c) Body of epididymis, sperm granuloma, HE 400x. (d) Ampullae of the vas deferens, diffuse interstitial and intraluminal inflammatory infiltrate, HE 200x.

Microscopic changes were similar between the two experimental infections. These changes were observed in 24.1% (49/203) and 28.7% (60/209) of all tissue samples from rams infected with *A. seminis* or *H. somni*, respectively. Immunohistochemical technique used was only effective for immunolabeling *A. seminis* in tissues. In the tail of the epididymis there was multifocal to diffuse chronic histio-lympho-plasmocytic interstitial infiltrate (Figures 7(a) and 8(a)), hyperplasia of the ductal epithelium, intraepithelial cysts (Figure 8(b)), and neutrophilic (microabscesses) or mixed intraepithelial and/or intraluminal infiltrate. Pyogranulomas composed of neutrophils, macrophages, epithelioid macrophages, and multinucleated giant cells, with central area of necrosis containing sperm and/or immunostained bacteria were also observed (Figures 7(c) and 7(d)). The lesions observed in the body of the epididymis were multifocal mononuclear interstitial or perivascular infiltrate and small spermatic granulomas (Figure 8(c)). Testicular changes were characterized by degeneration. There were 4/10 or 5/10 rams with left testicular degeneration in group infected with *A. seminis* or *H. somni*, respectively. Multifocal histio-lympho-plasmocytic interstitial infiltrate, neutrophilic intraepithelial infiltrate, and histiocytic and neutrophilic intraluminal infiltrate were observed in seminal vesicles (Figure 7). Immunostained* A. seminis* were observed within the cytoplasm of macrophages in the glandular lumen of seminal vesicles (Figure 7(f)). In ampullae of the vas deferens, interstitial and intraluminal inflammatory infiltrate (Figure 8(d)), mild to severe diffuse mononuclear interstitial infiltrate, multifocal neutrophilic intraepithelial infiltrate, and glandular hyperplasia were observed. Mild multifocal lymphohistiocytic infiltrate was observed in mucosa and submucosa of the urinary bladder. Lymphoid hyperplasia was observed in inguinal and iliac lymph node, as well as in the spleen from both experimental groups. The distribution, frequency, and intensity of the inflammatory changes in genitourinary tract are summarized in Tables 3 and 4.

## 4. Discussion

This is the first comparative study between *A. seminis* and *H. somni* experimental infections in rams, adding valuable information for a better understanding of the pathology and pathogenesis of these infections and, therefore, supporting further studies related to diagnosis of ovine infectious epididymitis. Although both agents are found in the preputial flora of healthy young sheep [5], *A. seminis* and *H. somni* can act as an opportunistic pathogen, causing primarily epididymitis and orchitis in young animals [6–8, 10–12]. So, this work demonstrated that the two organisms are capable of causing lesions in the reproductive tract of rams experimentally inoculated. Even with intraepididymal inoculation, both agents caused infection in different organs of genitourinarytract, affecting the urinary bladder, vas deferens, bulbourethral glands, and seminal vesicles. Additionally, in the case of* H. somni*, the kidneys and testes were also affected. However, none of the two agents demonstrated evidence of hematogenous dissemination, since they were not isolated from liver and spleen samples, and bacteremia was not detected at any time point during the course of experimental infections.

Clinical changes in the tail of epididymis that was inoculated were observed at 7 dpi in both groups. The increased length and width of the tails of the epididymides observed at 7 dpi in both experimental groups are in good agreement with previous studies [12, 23] and are compatible with an acute inflammatory process. According to previous studies, testicular and epididymal changes caused by inoculation of *A. seminis* may be noted at 1 dpi, whereas the changes are even more noticeable to clinical examination at 7 dpi [23]. Increase in scrotal circumference due to edema in the scrotum has been described only at 7 dpi infection with *A. seminis* [17]. Among clinical findings, we can also highlight the reduction in testicular consistency observed in both experimental groups, which was associated histologically to testicular degeneration. Severe testicular degeneration may impact directly on fertility of the animal. It is noteworthy that, in cases of chronic epididymitis, testicular consistency can increase irreversibly as a result of atrophy and interstitial fibrosis [8, 24]. The absence of inflammatory reaction and bacterial isolation from only two testes of all infected animals indicate that *A. seminis* and *H. somni* can indirectly compromise the testicles, inducing testicular degeneration due to epididymal inflammation. Similar changes have been observed in the testes of rams experimentally infected with *B. ovis* [16].

Gross and microscopic findings in both experimental infections were similar to those seen in cases of natural infection [3, 10, 11, 24–26]. Macroscopically, the changes observed more frequently in infected rams are abscess, usually located in the tail of the epididymis, thickening of the tunica vaginalis, fibrinous or fibrous periorchitis, and increase in size of seminal vesicles [1, 13]. The most severe microscopic lesions were observed at the site of inoculation (i.e., the tail of left epididymis), extending to the body of the epididymis. Microscopically, bacterial epididymitis initially elicits a neutrophilic inflammation that can be followed by epididymal epithelial hyperplasia or metaplasia, duct obstruction with content retention. Then ductal rupture can occur with extravasation of sperm, followed by diffuse fibrosis and granulomatous inflammation and formation of spermatic granulomas, as reported in this and other studies [8, 12, 16]. In addition to the epididymis, other organs with marked inflammation in this study included the left seminal vesicles and the vas deferens. A previous study has shown that rams inoculated with *A. seminis* directly in the epididymis may also develop vesiculitis, ampulitis, and bulbourethritis, in addition to epididymitis [8]. Díaz-Aparicio et al. (2009) [12] observed only epididymitis and ampulitis in rams experimentally infected *H. somni*. Importantly, vesiculitis is also commonly observed in rams infected with *B. ovis* [15, 16].

Shedding of *A. seminis* and *H. somni* occurred intermittently in the semen and urine, which is similar to what has been described in *B. ovis*-infected rams [21, 27]. It was confirmed that *A. seminis* and *H. somni* can survive well in the urinary tract, with the urine being an important source of elimination of causative agents of epididymitis, which supports its use for diagnosis of infectious ovine epididymitis [21, 28]. Interestingly, there was a low frequency of bacterial isolation at the inoculation site, which suggests that the chronicity of infection in the tail of the epididymis may prevent bacterial isolation [24].

## 5. Conclusions

Based on these results, we concluded that *A. seminis* and *H. somni* are capable of causing infection in rams by colonizing multiple organs of genitourinary tract. Furthermore, these two organisms induce indistinguishable clinical, gross, or microscopic findings. However, even without differentiating the etiological agent, clinical examination is essential as a screening procedure, because it allowed the detection of signs of infectious epididymitis. Conversely, as these infections are very similar, the importance of complementary diagnostic techniques is evident for confirmation of the etiologic agent in cases of ovine infectious epididymitis.

## Figures and Tables

**Table 1:** Frequency (%) of *Actinobacillus  seminis* and *Histophilus  somni* isolation from semen, blood, urine, and tissue samples from experimentally infected rams during six weeks of infection.

Sample	*Actinobacillus seminis *	*Histophilus somni *
Semen	70.0% (42/70)^a^	38.3% (23/70)^b^
Blood	0.0% (0/70)^a^	0.0% (0/70)^a^
Urine	60.0% (36/70)^a^	40.0% (24/70)^b^
Organs	17.1% (36/210)^a^	13.3% (28/210)^a^

^a,b^Different letters in the same line are results that differed by Fisher's exact test (*P* < 0.05).

**Table 2:** Frequency (%) of gross changes in rams experimentally infected with* Actinobacillus  seminis *or *Histophilus  somni*.

Macroscopic changes in infected sheep (*n* = 10)	*Actinobacillus seminis *	*Histophilus somni *
Abscess in the left epididymis tail	50%	50%
Abscess in the left epididymis body	20%	40%
Abscess in the right epididymis tail	10%	0%
Scrotal swelling	10%	0%
Fibrin in the pampiniform plexus	0%	30%
Fibrinous periorchitis	20%	30%
Hemorrhage adjacent left epididymis tail	20%	10%
Tunica vaginalis thickening with fibrous adhesion	40%	60%
Hypotrophy left testis	50%	10%
Increase in volume of vesicular glands	30%	0%
Increase in volume of the left urethral bulb	10%	10%
Inguinal lymphadenomegaly	60%	20%
Iliac lymphadenomegaly	50%	20%

**Table 3:** Distribution, frequency, and intensity of inflammatory lesions in the genitourinary organs of rams experimentally infected with *Actinobacillus  seminis*.

Tissues	Hl	Hr	Bl	Br	Tl	Tr	SVl	SVr	BUl	BUr	Am	Bd	Kd
Animals	1/10	0/10	4/9	0/10	9/9	1/10	5/9	1/10	1/10	0/10	5/10	5/10	1/10
Median*	0.2	0.1	1.0	0.1	2.4	0.3	1.4	0.3	0.2	0.1	1.1	0.6	0.3

*Inflammatory lesion score 0 = absent, 1 = mild, 2 = moderate, and 3 = severe. H: head of epididymis, B: body of epididymis, T: tail of epididymis, SV: seminal vesicle, BU: bulbourethral, Am: ampullae of vas deferens, Bd: bladder, Kd: kidney, l: left, and r: right.

**Table 4:** Distribution, frequency, and intensity of inflammatory lesions in genitourinary organs of rams experimentally infected with *Histophilus  somni*.

Tissues	Hl	Hr	Bl	Br	Tl	Tr	SVl	SVr	BUl	BUr	Am	Bd	Kd
Animals	2/10	3/10	5/10	0/10	9/10	0/10	6/10	0/10	3/10	2/10	5/10	4/10	2/10
Median*	0.2	0.4	1.0	0.0	2.1	0.1	1.4	0.2	0.6	0.6	1.1	0.6	0.3

*Inflammatory lesion score 0 = absent, 1 = mild, 2 = moderate, and 3 = severe. H: head of epididymis, B: body of epididymis, T: tail of epididymis, SV: seminal vesicle, BU: bulbourethral, Am: ampullae of vas deferens, Bd: bladder, Kd: kidney, l: left, and r: right.
